# Design and In Vivo Evaluation of an Intraocular Electrode for Ciliary Muscle Biopotential Measurement in a Non-Human Primate Model of Human Accommodation

**DOI:** 10.3390/bios15040247

**Published:** 2025-04-13

**Authors:** Sven Schumayer, Esmaeil Ghadiri Zahrani, Bahman Azarhoushang, Volker Bucher, Torsten Straßer

**Affiliations:** 1 Faculty Mechanical and Medical Engineering (MME), Institute for Microsystems Technology (iMST), Furtwangen University, 78120 Furtwangen, Germany; 2 Institute for Ophthalmic Research, University of Tuebingen, 72076 Tuebingen, Germany; torsten.strasser@uni-tuebingen.de; 3 Institute for Advanced Manufacturing (KSF), Furtwangen University, 78532 Tuttlingen, Germany; 4 Department of Microsystems Engineering (IMTEK), University of Freiburg, 79110 Freiburg, Germany; 5 University Eye Hospital Tuebingen, 72076 Tuebingen, Germany

**Keywords:** intraocular electrode, biopotential, electrode conception, accelerated aging, laser cutting

## Abstract

The measurement of electrical potentials in the human body is becoming increasingly important in healthcare as a valuable diagnostic parameter. In ophthalmology, while these signals are primarily used to assess retinal function, other applications, such as recording accommodation-related biopotentials from the ciliary muscle, remain poorly understood. Here, we present the development and evaluation of a novel implantable ring electrode for recording biopotentials from the ciliary muscle. Inspired by capsular tension rings, the electrode was fabricated using laser cutting, wiring, and physical vapor deposition coating. The constant impedance and weight over a simulated aging period of 391 days, demonstrated the electrode’s stability. In vivo testing in non-human primates further validated the electrode’s surgical handling and long-term stability, with no delamination or tissue ingrowth after 100 days of implantation. Recorded biopotentials from the ciliary muscle (up to 700 µV) exceeded amplitudes reported in the literature. While the results are promising, further research is needed to investigate the signal quality and origin as well as the correlation between these signals and ciliary muscle activity. Ultimately, this electrode will be used in an implanted device to record ciliary muscle biopotentials to control an artificial lens designed to restore accommodation in individuals with presbyopia.

## 1. Introduction

Since Galvani’s discovery of electrical activity in dissected skeletal muscle in 1786, followed by Matteucci’s demonstration in 1842 that frog heartbeats generate an electrical current, significant advances have been made in the field of bioelectrical recording. This culminated in Waller’s development of the electrocardiogram (ECG) in 1877, later refined by Einthoven, who was awarded the Nobel Prize in 1924 [1]. These milestones stimulated ongoing efforts to record electrical biopotentials from various tissues and organs throughout the body. The discovery of visually evoked potentials (VEP)—electroencephalographic (EEG) responses correlated with flashes of light—in the 1930s [2], triggered a growing interest in the study of biopotentials associated with vision and ocular function. Other methods, such as the electroretinogram (ERG) or the electrooculogram (EOG), to measure electrical activity in the eye were invented and soon used for research and diagnostics [3]. From the mid-1950s on, several research groups began recording electrical activity from the ciliary muscle [4,5,6,7,8,9,10,11]. This circular muscle surrounding the crystalline lens plays a key role in controlling accommodation [12], the process by which the eye focuses images on the retina. Despite these findings, interest in the ciliary muscle biopotentials waned, leaving a limited understanding. In other areas, biopotentials are now used not only for research and diagnosis, but also to control prostheses. This is achieved by recording the electrical activity of muscles using electromyography (EMG) [13,14,15] or neuronal signals for brain–computer interfaces using EEG or electrocorticography (ECoG) [16] in order to restore lost physical abilities. In recent years, interest in the ciliary muscle was revived by evidence of its involvement in the most common forms of ametropia, myopia, and presbyopia. Studies revealed differences in ciliary muscle morphology between myopes and emmetropes [17,18] and researchers demonstrated that ciliary muscle function remains intact in older adults despite the presence of presbyopia [19,20,21,22]. It could be said that age-related farsightedness is the most common form of a lost physical ability, affecting everyone beyond a certain age [23]. In order to develop a prosthesis for the future treatment of age-related farsightedness that is capable of restoring the accommodative feedback loop which is disrupted by the stiffening of the crystalline lens [12], it was necessary to develop an implantable intraocular electrode. Another approach would be to realize the visual aid in the form of a smart contact lens, but current concepts struggle to ensure sufficient energy density to power themselves due to space limitations [24,25]. Instead of implanting a battery as is currently performed, the power supply could be realized similar to retina [26] or cochlear implants [27,28]. While a contact lens-based approach would provide a non-invasive method of recording the ciliary muscle biosignals [5,6,7,9,11], it remains susceptible to recording artifacts caused by factors such as blinking and varies in signal amplitude due to imperfect positioning of the electrode in relation to the ciliary muscle. Needle electrodes, as used in early studies [7,9], on the other hand are invasive, damaging the ciliary muscle. In addition, they record spike trains of muscle activity rather than the summed potentials measured with the contact lens electrode. To overcome these merits, an intraocular bipolar ring electrode was developed that is designed to be placed in the *ciliary sulcus,* behind the iris, in front of the ciliary body. The design of the ring electrode was inspired by capsular tension rings that are routinely used to stabilize the capsular bag in the case of weakened or damaged zonula fibers [29] and in special cases, are also implanted in the *ciliary sulcus* [30]. This allows a minimally invasive implantation, a relatively large contact surface to the surrounding tissue, and eliminates the influence of artifacts such as blinking.

## 2. Materials and Methods

### 2.1. Electrode Design

**Figure 1:**
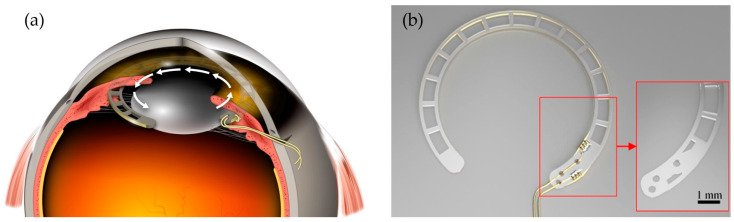
Ring-shaped bipolar electrode (**a**) Schematic drawing of a cross-section of the eye, where the ring electrode is inserted by an incision at the limbus and placed, by rotating (white arrow) it into the ciliary sulcus. (**b**) Rendering of the electrode. The inner and outer faces of the electrode are mainly connected by spokes. The magnification (red) highlights the geometry of the conducting area, where the gold wires are manually attached, before coating.

Given the similarities in accommodative apparatus, eye anatomy, and functionality between cynomolgus monkeys and humans [31,32], cynomolgus monkeys were selected as the preclinical model. The *sulcus* diameter in these non-human primates was determined theoretically and compared with measurements from explants. In this calculation, the equation provided by Mehdi et al. [33] for estimating the *sulcus* diameter in the human eye was used with values for the horizontal corneal diameter [34] and the corneal curvature [35], specific to cynomolgus monkeys, resulting in an outer diameter of 10 mm for the ring electrode. The bipolar electrode, made of polyethylenterephthalat (PET; Tekra, LLC; New Berlin; WI; USA), consists of two concentric rings connected by 1 mm-spaced spokes, allowing it to measure potential differences generated by the ciliary muscle during accommodation. The developed ring electrode leverages the concepts of the commercially available capsular tension rings (CTRs) and is implanted in the *sulcus ciliaris* behind the iris and in front of the ciliary body to keep the distance to the presumed target tissue as small as possible (Figure 1a). The geometry (Figure 1b) is a compromise between positioning, in terms of centering, contacting as well as implantability. In terms of biological safety (ISO 10993), only declared biocompatible materials were used, researched in the literature, and assured to withstand the selected overpressure ethylene oxide sterilization process.

### 2.2. Manufacturing—Laser Cutting

**Figure 2:**
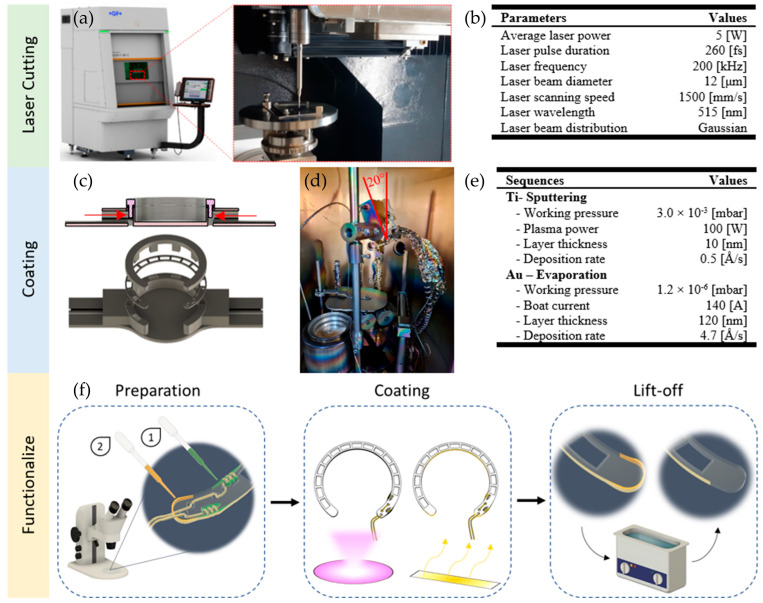
Manufacturing process of the ring electrode. (**a**) The 5-axis laser cutting machine with the magnified experimental setup within the machine, and the utilized laser parameters (**b**). (**c**) The “sandwich-like” masking, with the ring blank between. The cross-section shows the two channels (red arrows) where the gelling sugar is applied. (**d**) Setup in the physical vapor deposition (PVD) machine with the skewed (20°) sample holder and rotation unit above the shutter, which covers the gold boat. The silver round deepening at the lower left-hand side is the Ti-target. (**e**) The coating parameters of the ring electrodes for Ti as a bonding agent and Au as the electrode surface. (**f**) The schematic functionalization process, where first (1) the electrically conductive glue (green) is applied on the winded gold wires and cured at 80 °C for at least 10 h. Secondly (2), gelling sugar shown in orange, as a temporary masking agent, is applied on both tips of the ring electrode. Before evaporating 120 nm electrode material (Au), 10 nm Ti is sputtered as an adhesion promotor. The masking agent is dissolved within an ultrasonic cleaner for 3 min at 35 °C.

The layout was cut from a 200 µm thick PET film using a Carbide-Model laser (CB3-40-0200-10-HB; Light Conversion Company; Vilnius; Lithuania) with an initial emission wavelength of 1064 nm and a capability of second harmonic generation. The laser source was integrated into a 5-axis machine (LP400U; GF Machining Solutions; Geneva; Switzerland), as shown in Figure 2a. The intensity profile at the laser output was near-Gaussian (M^2^ < 1.2) and the beam spot size (2 w_0_) was at 12 µm. The laser provided a femtosecond pulse with a duration of 260 fs, at a wavelength of 515 nm and an average laser power of 5 W (cf. Figure 2b), assuring minimal thermal damage. The laser was guided six times along the designed contour on the film with a nominal constant focus distance of 100 mm. Subsequently, the blanks were examined using optical (VHX-700F; Keyence; Osaka; Japan) and scanning electron microscopy (XL30 ESEM; Phillips; Eindhoven; The Netherlands).

#### 2.2.1. Manufacturing—Electrode Surface Wiring Integration

Two 140 µm diameter perfluoroalkoxy (PFA)-coated gold wires (A-M Systems, Inc.; Carlsborg; WA; USA) were cut to a length of 45 mm and the ends (5 mm) were stripped. Using a digital microscope (VHX-700F; Keyence; Osaka; Japan), the ends were threaded through the holes used as strain reliefs until the insulated part of the wires reached the respective wedge-shaped cutouts. The stripped wire ends were manually wrapped three times around the wedge-shaped recesses to increase the bonding area with electrically conductive adhesive (EPO-TEK MED H20S; Epoxy Technology Inc; Billerica; MA; USA). The adhesive was selectively applied with a hand-held dispenser (THE-200; TAEHA Corporation, Namyangju-Si, Republic of Korea) at a pressure of 300 kPa for 0.3 s and manually distributed. Curing was performed in a climatic chamber (MKF115 E1.3; Binder GmbH; Tuttlingen; Germany) at 80 °C for at least 10 h to ensure that the adhesive was fully cross-linked. The ring electrode blank was then cleaned with a 40% IPA solution in an ultrasonic tank at 35 °C for 3 min. To avoid a continuous coating and thus, short-circuiting of the two electrode surfaces of the electrode, the biocompatible and masking material gelling sugar (2 plus 1; Suedzucker AG; Mannheim; Germany) was applied manually to the curves of the tips (cf. Figure 2f) and lifted off with an ultrasonic cleaner (RK 100H; Bandelin; Berlin; Germany) at 35 °C for 3 min after the coating process. Due to the upside-down position of the sample holder in the evaporation and sputtering unit (AUTO 306; HHV Ltd.; Crawley; UK), in which the conductive layers were to be applied, the 3D printed masking fixture was additionally equipped with two channels (Figure 2c). The wired blank was carefully placed on the upper part of the mask, which was then plugged together with the lower part and secured with gelling sugar injected into the channels (Figure 2c, red arrows). The entire mount was placed in a climate chamber at 60 °C and 5% humidity for 30 min to further reduce the moisture content of the gelling sugar.

#### 2.2.2. Manufacturing—Electrode Surface Coating

The blank was coated in an evaporation and sputtering system (AUTO 306; HHV Ltd.; Crawley; UK) with titanium (Ti) as an adhesion promoter, followed by gold (Au) as a conductive surface. Before deposition, a prepared sample plate and the connected rotation unit first had to be tilted 20° from the vertical position (cf. Figure 2d), to avoid shadowing effects. The gold wires were guided backward through a 3 mm drilling hole at the sample plate and covered with polyimide tape (3M; Saint Paul; MN; USA). Before sputtering, Ar-plasma cleaning for seven minutes was carried out with a glow discharge (3 kV; 50 mA; 8 × 10^−2^ mbar). The deposition parameters are shown in Figure 2e. During the entire process, the sample holder rotated to improve the homogeneity of the coating.

### 2.3. Testing—Estimated Post-Implantation Stability Through Accelerated Aging

Electrodes are exposed to a harsh environment due to body temperature, increased humidity, and the physiologically expected sodium concentration [36,37]. Therefore, the long-term stability of the ring electrodes (n = 4) was tested in a biosimilar environment using an accelerated aging test based on the ISO 10993-13 [38] standard and the protocol for accelerated aging [39]. The norm recommends choosing a test solution as similar as possible to the in vivo environment. As in previous studies [36,40,41], phosphate-buffered saline (PBS; 0.14 M NaCl, 2.7 mM KCl, 10 mM phosphate) with a pH-value of 7.4 was used (ROTI^®^Fair PBS 7.4; Carl Roth GmbH + Co.KG; Karlsruhe; Germany). The selected PBS solution matches the pH value of the aqueous humor (6.5–7.5 pH) in the anterior part of the eye [42]. The accelerated aging temperature is based on the maximum temperature (T_max_) of 60 °C recommended for testing polymers, at which it is guaranteed that the degeneration is primarily chemically driven according to the Arrhenius principle [39]. As specified in the standard ISO 10993-13, polypropylene tubes (50 mL tubes Cellstar^®^ 30/115 mm; Greiner Bio-One GmbH; Frickenhausen; Germany) with a volume ratio of at least 1 g sample to 10 mL test solution were selected. The minimum duration to be tested in days (R_test_) was determined by using the following equation:

(1) $$R_{Imp}=R_{test}\cdot Q_{10}^{\left(T_{max}-T_{Imp}\right)/10{}^{\circ}C}$$

The ambient temperature of the implant in vivo (T_Imp_) was determined to be 31.7 °C as a conservative estimate based on the temperature of the anterior chamber of the human eye of 29.9 ± 1.8 °C published in the literature [43]. The temperature coefficient (Q_10_) relative to 10 °C is based on empirical observations and is defined as Q_10_ = 2 for polymers used for medical applications [39]. The implantation period (R_Imp_) in the preclinical model was set at one year, resulting in a test period of 52 days. The samples were kept isolated on the heating plate over time and only taken for mass determination, microscopy, and electrochemical impedance spectroscopy according to the measurement protocol. Prior to mass determination on an analytical balance (Kern ADB 200-4; Kern & Sohn GmbH; Balingen; Germany), the samples were blown dry with a nitrogen jet and then further dried in the climate chamber for 30 min at 40 °C and 5% humidity. Immediately after the mass determination, electrodes were examined under a stereomicroscope (Stemi 508; Carl Zeiss Microscopy GmbH; Jena; Germany) and electrochemical impedance spectroscopy was performed. After measuring, the test container was rinsed first with isopropanol and then with deionized water before 15 mL of new PBS was filled in, and the sample was reinserted.

#### 2.3.1. Testing—Assessing Long-Term Electrical Stability via Electrochemical Impedance Spectroscopy

Electrochemical impedance spectroscopy (EIS) was performed to analyze the quality performance shift of the electrodes during the aging process. A two-electrode setup, electrically shielded in a Faraday cage, with a platinum wire (⌀ 0.5 mm) as a counter electrode (CE = 50.42 mm^2^) and the ring-shaped electrode as a working electrode (WE_inner_ = 3.519 mm^2^, WE_outer_ = 4.351 mm^2^) was placed in a 50 mL sample container filled with PBS (0.14 M NaCl, 2.7 mM KCl, 10 mM phosphate). A modified lid for the container ensured a distance of 35 mm between the counter electrode and the respective working electrode surfaces. Before measurement, the container was rinsed with isopropanol and distilled water. EIS was completed using a Solartron MaterialsLab XM (AMETEK Scientific Instruments; Berwyn; PA; USA) together with the XM-studios MTS Software (version 3.2). A sinusoidal voltage of 10 mV in a frequency range between 1 and 100 kHz, as recommended by Boehler et al. [37], was utilized as a stimulus with an offset voltage of 10 mV. Both electrode surfaces of the ring electrode were measured separately and analyzed using JMP^®^ (Version 16.0.0.; SAS Institute Inc.; Cary; NC; USA; 1989–2023).

#### 2.3.2. Testing—In Vivo Experiment

The bipolar ring electrode was implanted in the *ciliary sulcus* of the right eyes of two 7-year-old male cynomolgus monkeys to serve as an animal model for the study of human accommodation. The in vivo testing was performed at Labcorp Early Development Services GmbH (Münster, Germany) in accordance with Directive 86/609/EEC on animal experimentation. The study protocol was approved by the Landesamt für Natur, Umwelt und Verbraucherschutz (LANUV) of North Rhine-Westphalia, Germany (registration number 81-02.04.2023.A032).

The electrodes were double-packed in self-sealing sterilization pouches and sterilized by overpressure ethylene oxide sterilization. Each electrode was implanted in the right eye, by rotating it into the *ciliary sulcus*, behind the iris, through a 3 mm corneal tunnel incision less than 1 mm posterior to the *limbus* at the superior temporal quadrant.

Monkey 1 was euthanatized at 101 days. The eye was enucleated and preserved in a solution of 2% buffered formaldehyde and 2.5% glutaraldehyde to prevent tissue shrinkage [44]. On the day of pathology, the eye was bisected from the superior hemisphere anteriorly to posteriorly toward the optic nerve and examined under the microscope (VHX 5000, Keyence, Osaka, Japan). For histological analysis, the sample was embedded in paraffin, cut (5 µm thickness) every 450 µm, stained utilizing a hematoxylin/eosin solution, and examined under the microscope to assess tissue responses.

The biopotentials shown here were measured on the animal’s cage (monkey 2) and analyzed in JMP. A treat was presented to the animal and given after the corresponding change in focus. The top-level system architecture of the recording and transmission system and the associated specifications are explained separately [45]. In brief: the implant consists of a CR1025 lithium button cell battery (3V; 30 mAh), a flexible circuit board (2-layer) with an analog front end and a central control unit with a Bluetooth antenna, as well as the described bipolar ring electrode. Apart from the electrode, all components are coated with Parylene C (2 × 10 µm) using chemical vapor deposition (CVD) and partly covered in epoxy and silicone to increase mechanical stability while maintaining flexibility. Measurements are recorded with a sampling rate of 250 Hz, by a total signal-to-noise-and-distortion ratio (SNDR) of 56.7 dB, and an effective resolution of 3.64 µV. Details and additional data from these in vivo tests will be addressed in a subsequent publication.

## 3. Results and Discussion

The design of the intraocular electrode for recording electrical potentials of the ciliary muscle during accommodation was inspired by capsular tension rings used to stabilize the capsular bag in cases of zonular weakness during cataract surgery [29,46]. Commercially available capsular tension rings have a width of about 0.2 mm and are usually prestressed and unfold to their full-size during implantation. Although typically implanted in the capsular bag, they can also be positioned in the *ciliary sulcus* [30]. In this location, implants, such as those designed to measure intraocular pressure [47] or to treat glaucoma [48], have also been reported. We utilized the centric lateral forces of the capsular tension ring [29] to minimize the distance and to achieve optimal contact with surrounding tissue of the ciliary muscle. The shape of the ring electrode represents a balance between important factors such as centering, tissue contact, and ease of implantation. This concentric ring design of the electrode, connected by 1 mm spokes, allows for increased electrode surface area and curvature, which improves signal-to-noise ratio (SNR) by reducing impedance [49,50]. The electrode’s central placement within the *ciliary sulcus* minimizes the distance to the signal source and maximizes tissue contact, which is essential for capturing high-amplitude responses [51]. In addition, the design allows for temporary torsion during implantation, which facilitates surgical handling and ensures that the electrode, once in place, maintains optimal contact with the surrounding tissue. Unlike typical intraocular devices such as CTRs and intraocular lenses [29,46,52], which are often made of polymethylmethacrylate (PMMA) for its transparency and durability, polyethylenterephthalat (PET) was selected as the substrate for the electrode due to its superior chemical resistance to isopropanol used in manufacturing processes. PET, commercially available as Dacron^®^ is established as biocompatible and has a long history of safe use in ocular implants for iridocapsular and iris-fixation lenses in humans [53].

### 3.1. Electrode Fabrication

**Figure 3:**
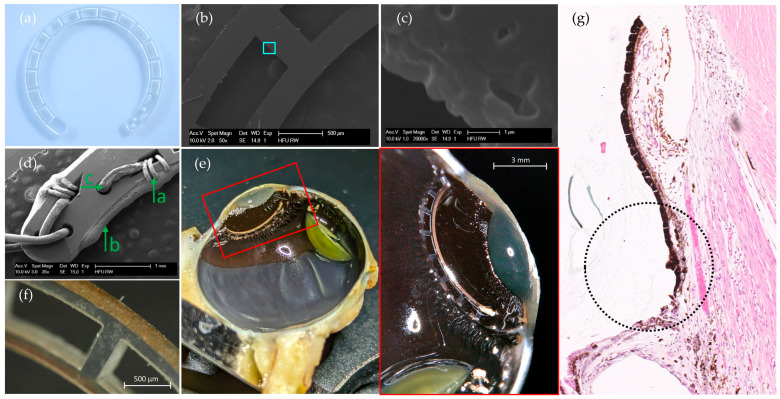
Microscope images. (**a**) The laser cut ring blank before connecting. (**b**,**c**) An edge at a spoke at different magnifications shows that there is a laser-induced melting zone of less than 3 µm. (Images (**b**–**d**) were taken with a SEM.) The light blue square shows the magnified area. (**d**) The wedge-shaped cut-out serves to temporarily fix the stripped gold wire (**d**-a) before it is bonded with an electrically conductive adhesive (**d**-b). The drill holes (**d**-c) serve as strain reliefs. (**e**) On the left-hand side, an overview of the left half of the enucleated eye (monkey 1) can be seen. The image on the right-hand side displays the magnification of the red box on the left-hand side and illustrates half of the electrode placed at the ciliary sulcus, anterior to the corona ciliaris, and posterior to the pupil. The yellow-greenish semi-circle is the lens, that slipped during cutting the eye. (**f**) An example of the outer, electrically conductive surface of the electrode #2 after the accelerated aging test. (**g**) Histological analysis of the ciliary sulcus (black dotted circle) in which the electrode was placed (cf. **e**). Above the circle is the posterior part of the iris and below, a base of the zonular fibers (ciliary process). The analysis, in the black dotted circle, shows a localized loss of ciliary body epithelium that may have occurred during surgery, although the ciliary body appears to be intact and unaffected.

The masking approach used in the coating process enables selective coating of the entire circumferential surface of round objects, providing a simpler and more cost-effective alternative to conventional planar methods. The process eliminates the need for photoresists and solvents, relying only on the mild isopropanol for a brief rinse, which helps preserve the chemical and mechanical properties of PET. The scanning electron microscope (SEM) images show a melt transition zone that could be minimized with optimized laser parameters; for example, the use of a telecentric f-theta lens could further reduce edge angle errors. The design flexibility, miniaturization potential, and production adaptability offered by laser cutting make it a promising fabrication technique for capsular tension rings or ring-shaped eye electrodes. Future adjustments to the spoke design, such as rounded edges, are expected to better distribute torsional forces within the electrode during implantation. Figure 3a shows the dimensional accuracy of the laser-cut blank at the microscopic level. The edges on the spokes, wedge-shaped recesses, and drill holes were precisely shaped according to the CAD model. Scanning electron microscope images (Figure 3b,c) confirm this accuracy, showing a melting zone of less than 3 µm. Figure 3d shows the manual electrode contacting, validating the production method described in the methodology.

#### 3.1.1. Long-Term Stability During Accelerated Aging

Considering the reproducibility of the scale (0.0002 g), the weight of the tested electrodes along the testing time was constant. Except electrode #4, which steadily decreased in weight, leading to a breakage during preparation for weighting on the seventh measurement sequence (cf. Appendix A). Visual inspection of the electrode surfaces showed no delamination nor other abnormalities.

The results suggest that three of the four electrodes would likely maintain mechanical stability over a simulated implantation period of 391 days. The weight of three electrodes remained constant, supporting their stability, with initial weight variability attributed to the manual application of conductive adhesive. The weight loss observed in the fourth electrode appears to be due to a fracture in one of its spokes, which may have been weakened during aging to the point of fracture. Alternatively, structural damage may have occurred during blow drying or handling. Some limitations of the test setup should be noted: Equation (1) is an approximation based on empirical data, considering only the temperature coefficient (Q_10_) and omitting the material-specific activation energy of aging-related reactions [54]. In addition, the use of PBS generalizes body conditions and the setup does not account for micromovements and oxidative stress that occur in vivo [40]. Nevertheless, the test complies with the standards of ISO 10993-13 [38] and demonstrates an implantation duration of at least one year, which is confirmed by in vivo results over a period of 101 days in monkey 1.

Electrical impedance spectroscopy was conducted in a two-electrode setup to measure the total impedance of the test configuration, including the counter electrode, working electrode, and electrolyte. Using a static measurement arrangement, it is possible to infer time-dependent changes in the working electrode. As expected, the PBS medium produced characteristic curves, typical of a parallel capacitor-resistor circuit. At lower frequencies, the electrode/electrolyte interface capacitance dominated, showing the anticipated inverse relationship with frequency and decreasing in influence as frequency increased. Accordingly, impedance decreased, and phase shifted upward.

**Figure 4:**
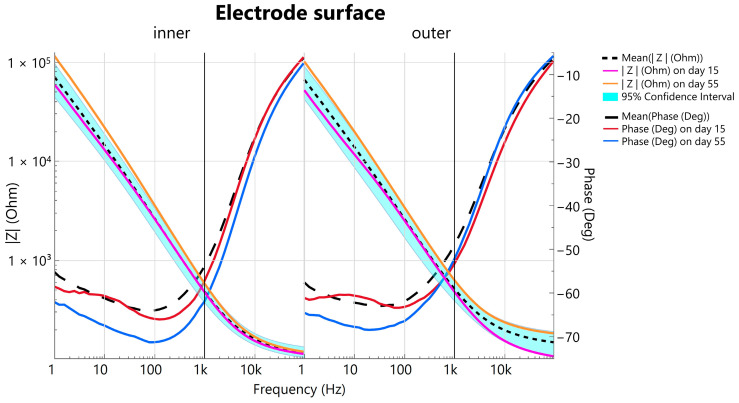
The electrochemical impedance spectroscopy. Bode plot of the inner (**left**) and the outer (**right**) electrode surface is shown up to day 55 equal to 391 days in vivo. Solid lines represent the mean, whereas the 95% confidence interval (cyan-colored area) is shaded. The dashed lines logarithmically represent the magnitude of impedance over the logarithmical scaled frequency range, whereas the long-dashed lines illustrate the phase shift over the frequency range.

A comparison of the narrow confidence interval of the impedance with measurements from the first to the 55th day (day: 1; 15; 22; 48; 55) shows that the impedance differs only slightly during accelerated aging (Figure 4). In addition, comparing the 1 kHz impedance |Z_1kHz_|, which can be taken as a benchmark parameter for electrode characterization [55] throughout simulated aging, the respective |Z_1kHz_| measurements on day 15 and day 55 are within the 95% confidence interval (cf. Figure 4—vertical line at 1 kHz) indicating a constant performance. The |Z_1kHz_| for the inner electrode surface is 483 ± 197 Ω (mean ± std.) and for the outer conductive surface, |Z_1kHz_| = 515 ± 227 Ω. Comparing the plots of the inner and outer electrode surface impedance and their phase shifts, a minimal difference is visible. The negative mean phase shift, which is the sum of the individual components in a parallel circuit, illustrates a predominant capacity behavior up to approximately 80 Hz for both surfaces which increases to −5.99° ± 1.9° (mean ± std.) for the inner electrode surface and to −5.98° ± 2.85° for the outer at 100 kHz, showing a diminishing capacity influence for high frequencies.

As described by Oldroyd et al. [40], who also investigated the stability of electrodes during an accelerated aging period, we declared an electrode as functional as long as the |Z_1kHz_| remained below 1 MΩ. Measurements with |Z_100kHz_| above 250 Ω were excluded as they indicate incorrect spacing of the electrodes and excessive contact resistance, which is why a direct comparison between day 1 and day 55 is not performed. On day 1, the electrodes were not yet in the PBS before the EIS measurement, but they were stored accordingly for the rest of the aging period. Although the electrodes were dried before the respective EIS measurement, deposits were still to be expected, which hypothetically influence the impedance as well as the phase. To mimic in vivo conditions, no further cleaning was performed before each impedance measurement. This may explain why the mean impedance at frequencies below 100 Hz on day 55 falls slightly outside the confidence interval.

For a discrete electrode characterization, a 3-electrode EIS setup would be required. Our objective of observing electrode stability over an accelerated aging period was successfully achieved. After simulating a one-year in vivo period (391 days), the electrodes maintained reliable functionality for detecting bioelectric potentials, with a mean |Z_1kHz_| of 583 Ω for the inner and 629 Ω for the outer electrode surface on day 55. The consistency of the |Z_1kHz_| values within the 95% confidence interval for days 15 and 55 highlights the stability of the electrode.

#### 3.1.2. Histology and In Vivo Recording

The bisected ring electrode can be seen in the anterior part of the sectioned eye of monkey 1 (Figure 3e). The electrode is intact after approximately three months (101 days) of implantation and is separated only at the site of the pathologic incision. There is no discoloration or opacification of the material nor any encapsulation. The inner electrode surface also shows no delamination, confirming the previously simulated results of accelerated aging (Figure 3f).

Histologic analysis of the eye of monkey 1, stained and examined microscopically (Figure 3g), revealed localized loss of ciliary body epithelium adjacent to the implanted ring electrode. This epithelial disruption is likely due to the mechanical action involved in positioning the electrode within the *ciliary sulcus* during implantation. While such minor epithelial changes are common with similar intraocular procedures and are generally not expected to interfere with device functionality, the literature notes that floating pigment particles resulting from chafing of an intraocular lens (IOL) in the *ciliary sulcus* have been associated with pigment dispersion syndrome, uveitis-glaucoma-hyphema (UGH) syndrome, iridocyclitis, and elevated intraocular pressure [56]. However, in this case, no significant adverse effects were observed, and the remaining ciliary structures and adjacent tissues appeared intact and unaffected, suggesting a low likelihood of complications from the epithelial disruption seen here.

**Figure 5:**
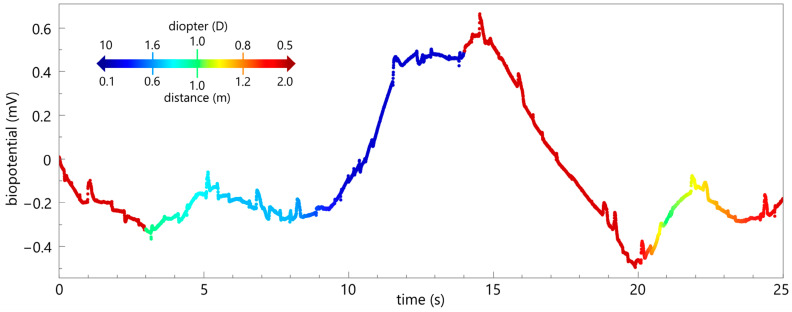
In vivo measurement. The raw measured biopotentials of the intraocular implant over a period of 25 s, with the approximate distance of the focus color-coded. The focus on the treat, which gradually moved towards the monkey, resulted in an increasing biopotential. A constant distance of about 15 cm (time: 11.5–14 s) caused a plateau-like shape while looking into the distance led to a voltage drop.

The short-term measurement in monkey 2 (Figure 5) demonstrates the functionality and the detected raw biopotential during change in focus. Approximately three seconds into the trial, a treat was presented at a distance of 1 m and approached by about 40 cm. This increased the measured biopotential by more than 0.2 mV. The treat was held at this distance (approx. 60 cm; time: 5–8 s) to allow the animal time to focus on it. The treat was then brought closer to about 15 cm and held at this distance for an additional 3 s. During this time, the detected voltage increased from −0.26 to 0.48 mV and formed a plateau (time: 11.5–14 s). At the 14 s mark, the animal shifted its gaze into the distance (>2 m), causing a voltage drop (−0.47 mV; time: 20 s). The animal refocused on the treat, which had been moved back to 1 m, resulting the voltage to increase to −0.13 mV (time: 22 s). After the short-term measurement, the treat was given to ensure positive reinforcement.

The short-term measurement is in agreement with the previous literature that measured accommodation-dependent biopotentials with a contact lens electrode [6,7,8,9,11] and plunge needle electrode [7,9]. They also describe the formation of a plateau during accommodation. The detected voltage change (≈0.7 mV) of the intraocular electrode is higher than the signals recorded with contact lens electrodes (up to 0.3 mV, c.f. Schubert et al. [6]) and, interestingly, higher compared with the needle electrode (up to 0.15 mV, cf. Hagiwara and Ishikawa, [9]). Due to the proximity to the origin, we expected higher amplitudes compared to the contact lens electrodes but not necessarily compared to the needle electrodes. A possible explanation is that the insertion of the needle electrode does not ensure correct positioning on the ciliary muscle if this is not additionally monitored. We are aware that the short-term measurement at this point represents only a moment in time and does not allow any comprehensive statements to be made about long-term functionality. In order to obtain detailed and reliable results, further investigations under controlled conditions are therefore in progress. Nevertheless, the data collected show that the characteristic, accommodation-dependent signal could be detected in vivo and the basic functionality of the developed electrode was successfully demonstrated.

## 4. Conclusions

In addition to the existing needle and contact lens electrodes, we have developed a new type of electrode that can be utilized to measure the biopotentials of the ciliary muscle. All steps from the design to the manufacturing of the electrodes along with the long-term tests in a body-like environment and the behavior of the electrode in vivo are addressed in this work. The electrode’s design is inspired by capsular tension, allowing it to be placed in the *ciliary sulcus* and combines mechanical flexibility with good electrical conductivity. Key design features include a concentric ring structure that increases surface area and curvature, thereby improving the signal-to-noise ratio, and a central placement within the *ciliary sulcus* for optimal tissue contact. Accelerated aging tests indicate that the electrode retains its functionality for a simulated implantation period exceeding one year. In vivo testing in a cynomolgus monkey model demonstrated the electrode’s capability to record biopotentials during accommodation, with recorded amplitudes exceeding those from prior contact lens and needle electrode designs. This work marks a promising advance toward an implantable device that could enable biofeedback control of an artificial lens, offering potential restoration of accommodation in presbyopia.

## 5. Patents

Sven Schumayer, Torsten Strasser, and Volker Bucher have patent #10 2024 116 619.3 pending to, Eberhard Karls Universität Tübingen Medizinische Fakultät. If there are other authors, they declare that they have no known competing financial interests or personal relationships that could have appeared to influence the work reported in this paper.

## Data Availability

The original dataset for measuring electrical impedance during the period of accelerated ageing, presented in this study, is openly available in FigShare under the following: DOI: 10.6084/m9.figshare.28497302.

## Abbreviations

The following abbreviations are used in this manuscript:

ECG	electrocardiogram
VEP	visually evoked potentials
ERG	electroretinogram
EOG	electrooculogram
EMG	electromyography
ECoG	electrocorticography
PET	polyethylenterephthalat
CTR	capsular tension ring
PFA	perfluoroalkoxy alkanes
IPA	isopropyl alcohol
Ti	titanium
Au	gold
Ar	argon
PVD	physical vapor deposition
PBS	phosphate-buffered saline
EIS	electrochemical impedance spectroscopy
CVD	chemical vapor deposition
SNDR	signal-to-noise-and-distortion ratio
SNR	signal-to-noise ratio
PMMA	polymethylmethacrylate
SEM	scanning electron microscope
CAD	computer-aided design
IOL	Intraocular lens
UGH	uveitis-glaucoma-hyphema

## Appendix A

	**Day**							
**Electrode**	**1**	**7**	**15**	**22**	**29**	**39**	**48**	**55**
#1	0.0150 g	0.0149 g	0.0149 g	0.0134 ^×^ g	0.0135 g	0.0134 g	0.0134 g	0.0134 g
#2	0.0146 g	0.0146 g	0.0146 g	0.0144 g	0.0145 g	0.0144 g	0.0144 g	0.0144 g
#3	0.0169 g	0.0169 g	0.0169 g	0.0168 g	0.0168 g	0.0169 g	0.0168 g	0.0168 g
#4	0.0151 g	0.0149 g	0.0148 g	0.0146 g	0.0145 g	0.0145 g	-	-
The change in weight in grams during the accelerated aging of each electrode, illustrated in tabular form. (^×^) A small piece of the cable was torn off when electrode #1 was improperly removed from the EIS measurement setup (day 22). Electrode #4 broke on a spoke on day 48 of accelerated aging testing and was not further considered for analysis.								
